# A Multi-Source Circular Geodesic Voting Model for Image Segmentation

**DOI:** 10.3390/e26121123

**Published:** 2024-12-22

**Authors:** Shuwang Zhou, Minglei Shu, Chong Di

**Affiliations:** 1 College of Computer Science and Engineering, Shandong University of Science and Technology, Qingdao 266590, China; ftdy123@outlook.com; 2 Shandong Artificial Intelligence Institute, Qilu University of Technology (Shandong Academy of Sciences), Jinan 250014, China; shuml@sdas.org

**Keywords:** geodesic voting, image segmentation, multi-source, polar representation, geodesic model

## Abstract

Image segmentation is a crucial task in artificial intelligence fields such as computer vision and medical imaging. While convolutional neural networks (CNNs) have achieved notable success by learning representative features from large datasets, they often lack geometric priors and global object information, limiting their accuracy in complex scenarios. Variational methods like active contours provide geometric priors and theoretical interpretability but require manual initialization and are sensitive to hyper-parameters. To overcome these challenges, we propose a novel segmentation approach, named PolarVoting, which combines the minimal path encoding rich geometric features and CNNs which can provide efficient initialization. The introduced model involves two main steps: firstly, we leverage the PolarMask model to extract multiple source points for initialization, and secondly, we construct a voting score map which implicitly contains the segmentation mask via a modified circular geometric voting (CGV) scheme. This map embeds global geometric information for finding accurate segmentation. By integrating neural network representation with geometric priors, the PolarVoting model enhances segmentation accuracy and robustness. Extensive experiments on various datasets demonstrate that the proposed PolarVoting method outperforms both PolarMask and traditional single-source CGV models. It excels in challenging imaging scenarios characterized by intensity inhomogeneity, noise, and complex backgrounds, accurately delineating object boundaries and advancing the state of image segmentation.

## 1. Introduction

Image segmentation is a fundamental task in computer vision, playing a crucial role in applications such as medical imaging [1,2], autonomous vehicles [3,4], and agriculture [5,6]. Given its wide-ranging applications, image segmentation has been extensively studied over the past few decades, resulting in the development of numerous methods aimed at solving this problem efficiently [7,8].

The advent of deep learning, particularly convolutional neural networks (CNNs), has led to significant advancements in image segmentation [9]. CNNs can automatically learn image features and contextual information from large-scale datasets, thereby markedly improving segmentation performance. Specific neural network architectures, such as U-Net [10], DeepLab [11], and PolarMask [12,13], are designed with multi-scale processing capabilities, enabling them to handle features and objects of varying scales simultaneously. However, existing neural network-based segmentation models primarily rely on local appearance and abstract features from convolutional layers based on regular image grids. These models often lack explicit consideration of geometric constraints and global object information, which could further enhance segmentation precision.

Meanwhile, researchers have also investigated energy functional-based active contour methods for image segmentation [14,15]. The core idea of these methods is to define an energy functional that accounts for specific image attributes, such as grayscale values, color information, and texture features. By employing optimization algorithms to minimize this energy functional, an optimal image segmentation result can be obtained. A notable advantage of active contour methods is their ability to design an energy functional based on the characteristics of the segmentation target. This approach naturally integrates prior information about the target, thereby enhancing model interpretability. However, a major limitation is that these methods do not automatically extract features from training samples. In practical applications, they often require manual or semi-automatic contour initialization based on test images and are highly sensitive to hyperparameters.

Given the limitations of neural network-based and active contour-based image segmentation methods, researchers have started to integrate these approaches to develop segmentation techniques with enhanced performance across various applications [16]. In the remainder of this section, we provide a brief overview of recent image segmentation methods that combine neural networks with active contour detection, followed by a discussion of the motivation behind our work.

### Integration of Neural Networks and Active Contour Models for Image Segmentation

In [17], the segmentation task is reframed as a contour detection problem, combining CNNs with active contour modeling to explicitly track contours without relying on dense intermediate predictions. Building on this integration, other studies have leveraged the synergy between CNNs and active contour methods for improved accuracy and robustness in diverse industrial and inspection tasks. For instance, the authors of [18] apply a lightweight depthwise CNN following an active contour-based segmentation stage to classify complex multi-defect wafer maps, achieving high accuracy under intricate manufacturing conditions. Similarly, the approach in [19] enhances deep learning-based contour detection by incorporating domain knowledge and specialized edge extraction to accurately delineate yarn boundaries, improving inspection performance in challenging textile environments.

Several methods have focused on refining local region modeling and parameter estimation within active contour frameworks. In [20], a local region-based active contour model employs Bayes’ theorem and spatial regularization to achieve robustness against noise when segmenting images with intensity inhomogeneity. Additionally, the authors of [21] combine CNN-generated parameter maps with texture descriptors, improving both speed and reliability during contour evolution. These techniques have shown particular success in medical imaging applications. For example, the authors of [22] fuse a modified active shape model with CNNs to accurately extract brain boundaries in MRI scans. In another instance, the authors of [23] integrate fuzzy connectedness with an autoencoder CNN, followed by post-processing with active contours, to enhance breast tumor segmentation in ultrasound images.

However, most of the existing approaches that integrate neural networks with active contour modeling still rely heavily on localized cues and lack a more global geometric understanding. In addition, many of these methods focus on specific applications, tailoring their designs and parameter settings to particular imaging scenarios and object classes, which limits their generalizability. These observations motivate the development of a method that not only leverages the strengths of deep learning but also integrates global geometric constraints to provide a more robust and general segmentation solution.

This paper proposes a novel approach that combines geometric constraints with neural networks. Specifically, it introduces a multi-source point circular geometric voting (CGV) method, termed PolarVoting. The PolarMask deep learning model [12] is utilized to extract feature representations at different scales and to obtain the primary contour of the object. Based on the center point and the source contour points identified by PolarMask, a new variant of the CGV model is proposed, where the voting score map is constructed using multiple source points. By leveraging the representational power of neural networks in conjunction with the global geometric information inherent in the image, the proposed PolarVoting method achieves precise and robust segmentation. It is particularly effective in complex imaging scenarios, accurately delineating object boundaries despite challenges such as intensity inhomogeneity, noise, and intricate background structures.

The contributions of this paper are summarized as follows:

1. We introduce a variational PolarVoting model for image segmentation that blends the enhancement from the geometric regularization of the CGV approach and the learning capabilities of the deep learning-based PolarMask model. This fusion leverages the classical PolarMask model to generate initialization information, thereby enhancing segmentation precision, especially in complicated scenarios.
2. The traditional CGV model utilizes a single user-provided source point to create the voting score map, which may suffer from unexpected difficulties for complex images. Our model advances the CGV model by incorporating multiple source points, an enhancement designed to achieve more robust segmentation, particularly for intricate object boundaries.
3. The efficacy of the proposed PolarVoting model is evaluated on both real-world natural and medical images. Experimental results demonstrate that the PolarVoting model indeed outperforms both PolarMask and the traditional single-source CGV model, underscoring its effectiveness across varied contexts.

## 2. Preliminaries

In this paper, we propose the integration of the PolarMask deep learning model [12,13] with the CGV method [24], thereby leveraging the representational capabilities of deep neural networks alongside the geometric regularization inherent in geodesic models. This section introduces the core principles underlying the PolarMask model and the CGV model, as well as a discussion of their respective limitations, which serve as the impetus for the development of the novel image segmentation model presented in this paper.

### 2.1. PolarMask Model

The PolarMask model is a widely recognized single-shot instance segmentation framework that predicts image masks in polar coordinates both effectively and efficiently. This approach has demonstrated promising performance in object image segmentation tasks. First, we present the overall framework of PolarMask, followed by a discussion of its limitations.

#### 2.1.1. PolarMask Segmentation

**Figure 1:**
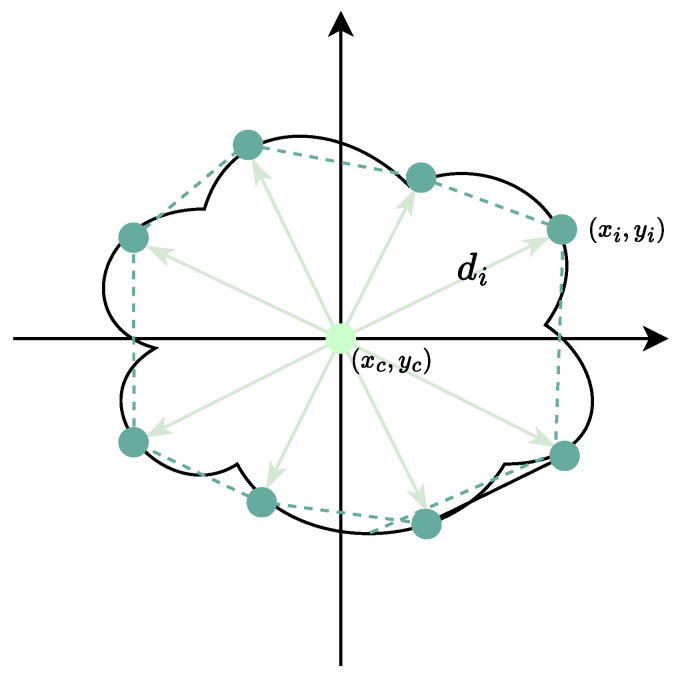
Illustration for a polar representation of a mask. The light green dot denotes the center point $\left(x_{c},y_{c}\right)$ and the dark green dots are the sampled boundary points $\left(x_{i},y_{i}\right)$ for 1≤i≤n.

As shown in Figure 1, the PolarMask model introduces an efficient way to represent the image segmentation mask using the polar coordinate system, where an instance mask is represented by a single center and *n* straight rays. For a given instance mask, PolarMask first samples a candidate center $\left(x_{c},y_{c}\right)$ of the instance and a set of boundary points $\left(x_{i},y_{i}\right)$ for i=1,2,…,n located on the predicted contour. From the center point $x_{c},y_{c}$, *n* rayline-like straight segments are emitted uniformly at equal angular intervals Δθ, with their lengths determined by the distance from the center point to corresponding boundary point $\left(x_{i},y_{i}\right)$. The angular resolution is predefined (e.g., $n=8,\;\Delta \theta =45^{\circ }$). The length *r* of each rayline-like segment is predicted through a learning process. As a consequence, the image segmentation consists of an instance center classification processing and a dense distance regression processing in polar coordinates. With the polar coordinate representation, the PolarMask mask incorporates the polar IoU loss and soft polar centerness for instance center classification and dense coordinate regression, which further enhances its performance. For more detailed information, we refer the reader to [12,13].

The PolarMask model integrates a backbone network, a modified feature pyramid network, and a task-specific head [25]. Given the center location $\left(x_{c},y_{c}\right)$ and the lengths of *n* rays, the position of each corresponding contour point $\left(x_{i},y_{i}\right)$ is calculated by:

(1) $$x_{i}=\cos\theta_{i}\times d_{i}+x_{c}$$

and

(2) $$y_{i}=\sin\theta_{i}\times d_{i}+y_{c}.$$

Starting from the angle $0^{\circ }$, the boundary points are sequentially connected, ultimately forming the complete contour and the corresponding mask.

#### 2.1.2. Limitations of the PolarMask Model

**Figure 2:**
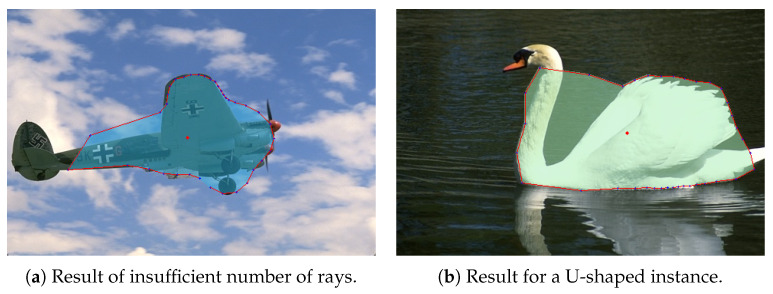
Result in challenging scenarios.

The PolarMask model offers a novel approach to single-shot image segmentation, significantly improving performance and simplifying the training process. However, PolarMask still faces significant challenges when addressing image segmentation tasks, particularly in the following three scenarios:

1. The PolarMask model constructs the final contour by directly connecting the predicted points on the instance contour. This means that to achieve a more detailed representation of the contour, a greater number of polar rays must be predicted. For instance, as shown in Figure 2a, constructing the contour of an instance using only 36 points would lead to poor segmentation results. Furthermore, as previously mentioned, the mask regression branch has a shape of H×W×n to predict the length of each polar ray. When the number of rays is large, the branch structure becomes more complex, making training more challenging. Therefore, selecting an appropriate value for the hyper-parameter *n* is a delicate task for PolarMask.
2. In the PolarMask model, only the point with the greatest length for each ray is preserved. However, if there are multiple points along the ground truth contour, as in the case of the example shown in Figure 2b, PolarMask struggles to accurately capture the true contour of the instance, even when the number of rays is large. As a result, PolarMask encounters significant challenges in segmenting U-shaped instances.
3. The PolarMask model does not incorporate contour regularization. In scenarios where image quality is low or distorted by noise, such as in medical CT images, the absence of regularization can lead to unexpected and irregular segmentation results, highlighting significant room for improvement.

### 2.2. The Circular Geodesic Voting Model

The circular geodesic voting model (CGV) [24] is a novel variational model designed to address the interactive image segmentation problem, particularly effective for complex tasks such as medical image segmentation. This section introduces the key concepts underlying the method and discusses the limitations that this paper aims to address.

#### 2.2.1. Geodesic Minimal Path Model with Asymmetric Quadratic Metrics

The general geodesic minimal path problem is defined on a bounded domain $\Omega \subset R^{n}$ equipped with a metric F(x,u) that depends on positions x∈Ω and orientations $u\in R^{n}$. This metric F defines a norm at each point x as:

(3) $$F_{x}\left(u\right):=F\left(x,u\right),$$

where the norms $F_{x}$ must be positive $F_{x}\left(u\right)>0$ whenever u≠0, 1-homogeneous, and satisfy the triangle inequality. However, in general, these norms are allowed to be asymmetric:

(4) $$F_{x}\left(u\right)\neq F_{x}\left(-u\right).$$

The length of a regular curve γ with respect to the metric F is measured as:

(5) $$L_{F}\left(\gamma \right)=\int_{0}^{1}F\left(\gamma \left(t\right),\gamma^{\prime }\left(t\right)\right)\;dt.$$

The minimal action map U(x) is defined as:

(6) $$U\left(x\right):=\inf\{L_{F}\left(\gamma \right);\;\gamma \in A_{s,x}\},$$

where $A_{s,x}$ represents the collection of all Lipschitz paths γ:[0,1]→Ω such that γ(0)=s and γ(1)=x. This map is the unique viscosity solution to an Eikonal partial differential equation (PDE) [26,27]:

(7) $$\left\{\begin{array}{cc} F_{x}^{*}\left(\nabla U\left(x\right)\right)=1, & \forall x\in \Omega \setminus \{s\},\\ U(s)=0, \end{array}\right.$$

where $F_{x}^{*}$ is the dual norm of $F_{x}$, defined for all $u\in R^{n}$ by:

(8) $$F_{x}^{*}\left(u\right):=\underset{v\neq 0}{\sup}\frac{\langle u,v\rangle }{F_{x}\left(v\right)}.$$

Based on the definition of the dual norm in (8), the corresponding optimal direction map Ψ is obtained by:

(9) $$\Psi \left(x,u\right):=\arg\max_{v\neq 0}\frac{\langle u,v\rangle }{F_{x}\left(v\right)},\;\forall x\in \Omega ,\;\forall u\in R^{n}.$$

The geodesic $C_{s,x}$ is obtained by reversing the geodesic ${\hat{C}}_{x,s}$ with $C_{s,x}\left(0\right)=s$ and $C_{s,x}\left(1\right)=x$, where ${\hat{C}}_{x,s}$ is tracked through the following ordinary differential equation (ODE) involving the minimal action map *U* and the optimal direction map Ψ:

(10) $$\left\{\begin{array}{c} {\hat{C}}_{x,s}^{\prime }\left(t\right)\propto -\Psi \left({\hat{C}}_{x,s}\left(t\right),\nabla U\left({\hat{C}}_{x,s}\left(t\right)\right)\right),\\ {\hat{C}}_{x,s}\left(0\right)=x. \end{array}\right.$$

Numerically, the ODE expressed in Equation (10) is solved using methods such as Heun’s or Runge–Kutta’s, or more robustly with the numerical method proposed by [28].

The asymmetric quadratic metrics $F_{AQ}$ used in the geodesic minimal path model within CGV are defined by:

(11) $$F_{x}:=F_{AQ}\left(x,u\right)=\sqrt{\langle u,M\left(x\right)u\rangle +{\langle \omega \left(x\right),u\rangle }_{+}^{2}},$$

where $M:M\to S_{2+}^{+}$ is a tensor field, $\omega :M\to R^{2}$ is a vector field, and ${\langle u,v\rangle }_{+}^{2}={\left(\max\left\{0,\langle u,v\rangle \right\}\right)}^{2}$ with $u,v\in R^{2}$.

To leverage edge asymmetry features, two anisotropic and asymmetric minimal paths can be traced from a source point to an endpoint on the target edge along two opposite edge tangent directions based on the asymmetric quadratic metric. A closed contour is then formed by combining these two minimal paths, which share the same source and target points, to delineate the target boundary. For this purpose, the asymmetric quadratic metrics, constructed with different rotation matrices $M_{i}$ (i∈{0,1}) and vector fields $\omega_{i}$ in the second part of the metric, are used to track minimal paths in counter-clockwise and clockwise directions. The corresponding asymmetric quadratic metrics are defined as:

(12) $$F_{AQ}^{0}\left(x,u\right)=\sqrt{\langle u,M\left(x\right)u\rangle +{\langle \omega_{0}\left(x\right),u\rangle }_{+}^{2}},$$

(13) $$F_{AQ}^{1}\left(x,u\right)=\sqrt{\langle u,M\left(x\right)u\rangle +{\langle \omega_{1}\left(x\right),u\rangle }_{+}^{2}}.$$

Unlike the isotropic metric, which is independent of path tangents, the asymmetric quadratic metrics can generate different minimal paths with the same source point and endpoint by reversing the vector field component.

#### 2.2.2. Overall Pipeline

The overall pipeline of the CGV model includes the following four components: computing the adaptive cut, building the set of endpoints, performing geodesic voting, and final segmentation.

##### Computing the Adaptive Cut

The adaptive cut [15] is integrated into the segmentation framework in conjunction with the minimal path model associated with the asymmetric quadratic metric. The use of the adaptive cut helps avoid the shortcut problem by preventing minimal paths from crossing the adaptive cut.

The adaptive cut is generated by computing a minimal path that connects a landmark point *z* inside the target region to a point *x* on the image boundary. It is constructed based on the Cohen–Kimmel minimal path model, where the metric in Equation (5) is simplified as $F_{\mathrm{Iso}}\left(x,u\right)=\psi \left(x\right)∥u∥$. The potential function ψ is designed so that the resulting minimal path intersects the target boundary only once. The geodesic distance map $U_{z}:M\to R_{0}^{+}$ can be computed by solving the PDE (7) associated with the metric $F_{\mathrm{Iso}}$.

The endpoint b∈∂M of the target adaptive cut $C_{z}$ can be identified by finding the point with the minimum distance value, i.e.,

(14) $$b:=\arg\min_{x\in \partial M}U_{z}\left(x\right).$$

Finally, the adaptive cut $C_{z}$ can be generated by solving the gradient descent ODE (10) on $U_{z}$ such that $C_{z}\left(0\right)=p$ and $C_{z}\left(1\right)=b$. Since the adaptive cut intersects the target boundary only once, it allows for tracking the minimal path from one side of the cut to the other.

##### Building the Set of Endpoints

The farthest point sampling (FPS) scheme [29] is employed to generate a set of points $A=\{p_{k}\in M;\;1\leq k\leq N\}$ with N≥3, which is then used to construct the set of endpoints for computing anisotropic and asymmetric minimal paths.

All points in the adaptive cut $C_{z}$ are utilized as the initial model to search for the farthest points. The set of sampled farthest points is denoted as P. In the first iteration, P is set as $P^{\left(0\right)}=C_{z}$, where $P^{k}$ represents the updated set at the *k*-th iteration. The first farthest point $p_{1}$ is detected via the corresponding geodesic distance map $U_{C_{z}}$ with $P=P^{\left(0\right)}$, as follows:

(15) $$p_{1}=\arg\max_{x\in M}U_{P}\left(x\right).$$

The set P is then updated to $P^{\left(1\right)}=P^{\left(0\right)}\cup \left\{p_{1}\right\}$. In the *k*-th iteration (where 1<k≤N), the *k*-th farthest point $p_{k}$ is detected by:

(16) $$p_{k}=\arg\max_{x\in M}U_{P}\left(x\right).$$

This process yields the updated set $P^{\left(k\right)}=P^{\left(k-1\right)}\cup \left\{p_{k}\right\}$. Finally, the set of farthest points *A* is generated as:

(17) A=P∖C.

##### Performing Geodesic Voting

Using the sampled endpoints, a set of minimal paths for constructing the voting score map can be established by connecting each endpoint in the farthest point set *A* with asymmetric quadratic paths, based on the asymmetric quadratic metric. All minimal paths originate from the source point, which is the intersection of the target boundary and the adaptive cut. Let $\Phi_{AQ}={\left\{G_{i,j}\right\}}_{1\leq i\leq N}^{j\in \{0,1\}}\subset \mathrm{Lip}\left(\left[0,1\right],M\right)$ represent the set of voting paths, where *N* is the total number of endpoints. For each endpoint $p_{i}\in A$, two minimal paths, $G_{i,0}$ and $G_{i,1}$, corresponding to the metrics $F_{AQ}^{0}$ and $F_{AQ}^{1}$, are computed from the initial point *s* along both sides of the adaptive cut. The geodesic distance maps $U_{i,0}$ and $U_{i,1}$ are calculated using the adapted Hamiltonian fast marching (HFM) method in conjunction with the adaptive cut.

For an endpoint $p_{i}$, the minimal paths $G_{i,0}$ and $G_{i,1}$ are obtained with respect to the geodesic distance maps $U_{i,0}$ and $U_{i,1}$. The minimal path $G_{i,j}$ with the lower weighted curve length is selected for voting score construction, as it has a higher likelihood of accurately depicting the target boundary. In this context, the weighted lengths of $G_{i,0}$ and $G_{i,1}$ are $U_{i,0}\left(p_{i}\right)$ and $U_{i,1}\left(p_{i}\right)$, respectively. The path with the lower length between $G_{i,0}$ and $G_{i,1}$ is denoted by ${\hat{G}}_{i}$, where ${\hat{G}}_{i}=G_{i,0}$ if $U_{i,0}\left(p_{i}\right)<U_{i,1}\left(p_{i}\right)$, and ${\hat{G}}_{i}=G_{i,1}$ otherwise. For convenience, define:

(18) $$\Phi_{D}={\left\{{\hat{G}}_{i}\right\}}_{1\leq i\leq N}\;.$$

The voting score map $V^{D}:M\to \left[0,\infty \right)$, associated with the initial point *s*, is defined as:

(19) $$V^{D}\left(x\right)=\sum_{G\in \Phi_{D}}\chi_{X}\left(G\right),$$

where $\chi_{X}:\mathrm{Lip}\left(\left[0,1\right],\Omega \right)\to \left\{0,1\right\}$ is a path detector defined by:

(20) $$\chi_{X}\left(\gamma \right)=\left\{\begin{array}{cc} 1, & \exists t\in [0,1]\;\mathrm{such}\;\mathrm{that}\;\gamma (t)=x,\\ 0, & \mathrm{otherwise}. \end{array}\right.$$

The value $\chi_{X}\left(\gamma \right)$ equals 1 if the curve γ passes through the point x, and 0 otherwise.

For each pair of minimal paths $G_{i,0}$ and $G_{i,1}$ leading to $p_{i}\in A$, these paths are considered to form a closed contour that accurately depicts the target if the geodesic flows at the endpoint are inverse to each other. The Euclidean scalar product of the vector fields at point $p_{i}$, derived from the geodesic flow maps $V_{i,0}$ and $V_{i,1}$, is expressed as:

(21) $$T\left(p_{i}\right)=\frac{\langle V_{i,0}\left(p_{i}\right),V_{i,1}\left(p_{i}\right)\rangle }{∥V_{i,0}\left(p_{i}\right)∥∥V_{i,1}\left(p_{i}\right)∥},$$

with $G_{i,0}\left(1\right)=G_{i,1}\left(1\right)=p_{i}$. Given a threshold ζ∈[−1,0), the minimal paths $G_{i,0}$ and $G_{i,1}$ linked to $p_{i}$ are regarded as forming a valid closed path if $T(p_{i})<\zeta$. Let $\Phi_{F}={\left\{G_{n,j}\right\}}_{1\leq n\leq M,0\leq j\leq 1}$ denote the set of all minimal paths from $\Phi_{AQ}$ that satisfy this condition, where M∈(0,N] is a positive integer representing the number of selected minimal paths. The second voting score map $V^{F}:M\to \left[0,\infty \right)$ is then established as:

(22) $$V^{F}\left(x\right)=\sum_{G\in \Phi_{F}}\chi_{X}\left(G\right).$$

The final voting score map V:M→[0,∞), associated with the initial point *s*, is defined as:

(23) $$V\left(x\right)=\alpha V^{F}\left(x\right)+\beta V^{D}\left(x\right),$$

where the weight parameters α and β control the relative importance of the two voting score maps.

##### Final Segmentation

In the voting score map, a high value of V(x) indicates a strong likelihood that x belongs to the target boundary. A thresholding procedure is then applied to the voting score map V to obtain the initial segmentation results. Additionally, mathematical morphological operators are employed to refine these results, ensuring that the final contours have a width of a single grid point.

#### 2.2.3. Limitations

The CGV model demonstrates significant advantages by integrating the disconnection constraint within the image domain with the anisotropic and asymmetric properties of image edges. This approach allows for the detection of boundary contours of complex objects using only a randomly placed landmark point within the target region. Despite these strengths, the model has certain limitations that warrant further consideration:

1. The CGV model is inherently designed for interactive image segmentation, as it relies on the manual input of a landmark point. Consequently, it is unsuitable for scenarios requiring fully automatic segmentation.
2. In constructing the score map, the model utilizes a single point on the instance contour as the source point. While effective for simpler contours, this approach may struggle to accurately capture the complexity of highly intricate instance contours.

## 3. A Multi-Source Circular Geodesic Voting Model

In this section, we present the core contribution of this work: integrating the geometric properties of the CGV approach with the learning capabilities of the deep learning-based PolarMask model. This integration enhances the traditional CGV model by incorporating multiple source points to improve the computation of the voting score map.

**Figure 3:**
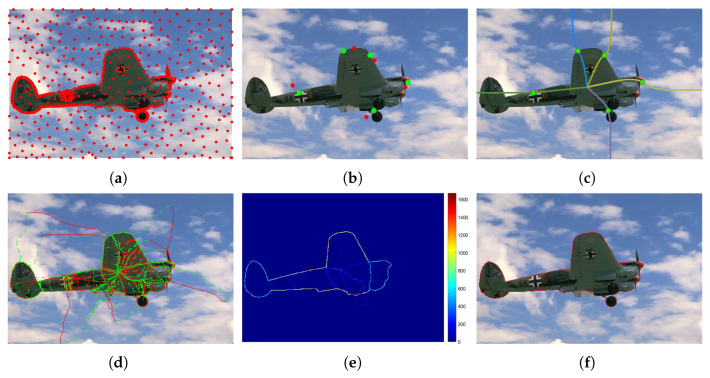
Overview of the proposed PolarVoting framework. (**a**) The original image, where the red dots represent the sampled farthest points. (**b**) Updating of vertices: the red dots denote contour points obtained from PolarMask, while the green dots represent the updated points. (**c**) Construction of multiple adaptive cuts. (**d**) Visualization of minimal paths using different metrics. (**e**) Visualization of the voting score map. (**f**) The final segmentation contour, represented by the red line.

An overview of the proposed PolarVoting framework is shown in Figure 3. The method involves two primary steps: (i) updating the vertices, which are derived from the PolarMask model and located near the target boundary, and (ii) computing a series of continuous curves, each of which passes through a vertex and connects the center point to the image domain boundary.

### 3.1. Update the Vertices

Suppose that the output from the classical PolarMask model is a set of landmark points ${\left\{{\tilde{p}}_{j}\right\}}_{1\leq j\leq J}$, where *J* is a positive integer. We further suppose that these points are distributed along the target boundary in a clockwise order. However, these points sometimes are not exactly located in the target boundary. In order to solve this problem, we move these points towards the close edge points identified using the image gradient magnitude. Specifically, let $I=\left(I_{1},I_{2},I_{3}\right):\Omega \to R^{3}$ be a vector-valued image (color image), where $I_{k}$ for k=1,2,3 represent the respective channel of the color image. As introduced in [30], the gradients of the color image I, denoted by ∇I, are defined in its Jacobian matrix, that is

(24) $$\nabla I\left(x\right)=\left[\begin{array}{c} \nabla G_{\sigma }*I_{1},\;\nabla G_{\sigma }*I_{2},\;\nabla G_{\sigma }*I_{3} \end{array}\right],$$

where $\nabla G_{\sigma }=\left(\partial_{a}G_{\sigma },\partial_{b}G_{\sigma }\right)$ are the standard euclidean gradients of the Gaussian kernel with deviation σ and $\partial_{a}G_{\sigma }$ (resp. $\partial_{b}G_{\sigma }$) is the partial differential of $G_{\sigma }$ along *a*-axis (resp. *b*-axis). Then, we construct a symmetric quadratic definite matrix field

(25) $$W\left(x\right)=\nabla I\left(x\right)\nabla I{\left(x\right)}^{⊤}$$

whose eigenvalues are $\lambda_{1}\left(x\right)$ and $\lambda_{2}\left(x\right)$ with $\lambda \left(x\right)\leq \lambda_{2}\left(x\right)$, such that we define $f:\Omega \to R_{0}^{+}$ as the image edge indictor such that

(26) $$f\left(x\right)=\lambda_{1}\left(x\right)+\lambda_{2}\left(x\right).$$

In essence, high values of the function f(·) usually imply strong appearance of image edges. As a consequence, we consider rectifying the landmark points ${\left\{{\tilde{p}}_{j}\right\}}_{1\leq j\leq J}$ by detecting an optimal point, denoted by $p_{j}$, in a neighborhood $T_{\xi }\left({\tilde{p}}_{j}\right)$ which surrounds the original landmark point ${\tilde{p}}_{j}$, where $\xi \in R^{+}$ is a positive scalar value defining the size of $T_{\xi }\left({\tilde{p}}_{j}\right)$.

In this work, this procedure involves two main steps, where the first one is the construction of the neighbourhood $T_{\xi }\left({\tilde{p}}_{j}\right)$. In order to take into account the image data, we apply the minimal weighted curve length associated to each point ${\tilde{p}}_{j}$ to recover the small region $T_{\xi }\left({\tilde{p}}_{j}\right)$. Specifically, this is implemented by estimating the following map of minimal weighted curve length

(27) $$D_{{\tilde{p}}_{j}}\left(x\right)=\min_{\gamma_{{\tilde{p}}_{j},x}}\int_{0}^{1}R\left(\gamma_{{\tilde{p}}_{j},x}\left(u\right),\gamma_{{\tilde{p}}_{j},x}^{\prime }\left(u\right)\right)\;du$$

where $\gamma_{{\tilde{p}}_{j},x}:\left[0,1\right]\to \Omega$ is a curve of Lipschitz continuity, subject to $\gamma_{{\tilde{p}}_{j},x}\left(0\right)={\tilde{p}}_{j}$ and $\gamma_{{\tilde{p}}_{j},x}\left(1\right)=x$. Let $\lambda_{i}$ (i=1,2) be the eigenvalues of the matrix field *W* as defined in Equation (25). In addition, let $r:\Omega \to R^{2}$ be a vector field such that the direction r(x) points from x to the closest image edge. For this purpose, we apply the method [31] to construct such a vector field r and we further suppose that $r_{⊥}\left(x\right)$ is a vector perpendicular to r(x). The function $R:\Omega \times R^{2}\to \left[0,\infty \right]$ is a standard Riemannian metric expressed as

(28) $$R\left(x,u\right)=r_{⊥}\left(x\right)r_{⊥}{\left(x\right)}^{⊤}+\exp\left(\mu f\left(x\right)\right)r\left(x\right)r{\left(x\right)}^{⊤}$$

where μ>0 is a positive constant. The computation of the minimal weighted curve length $D_{{\tilde{p}}_{j}}$ can be efficiently estimated using the Hamiltonian fast marching method [28,32]. Moreover, each point x leads to a minimal path $\gamma_{{\tilde{p}}_{j},x}^{*}$, whose Euclidean length is $L\left(\gamma_{{\tilde{p}}_{j},x}^{*}\right)$. Then, we define the neighbourhood $T_{\xi }\left({\tilde{p}}_{j}\right)$ as

(29) $$T_{\xi }\left({\tilde{p}}_{j}\right)=\left\{\forall x\in \Omega ,\;L\left(\gamma_{{\tilde{p}}_{j},x}^{*}\right)\leq \xi \right\}.$$

Eventually, the rectified points $p_{j}$ can be obtained by

(30) $$p_{j}=\underset{x\in T_{\xi }\left({\tilde{p}}_{j}\right)}{\arg\max}\;f\left(x\right).$$

An example of updating five PolarMask contour points is illustrated in Figure 3c.

### 3.2. Construct Multiple Adaptive Cuts

Now, we have a set of rectified landmark points ${\left\{p_{j}\right\}}_{j}$ distributed along the boundary of the target and a center point c inside the target. In contrast to the adaptive circular geodesic model [15] whose adaptive cut only connects the center point to a point at the image domain boundary ∂Ω, the introduced method is able to simultaneously pass through a specified point such as a landmark point $p_{j}$, allowing us to blend the benefits from the landmark points and the circular minimal paths. We denote by $C_{j}$ as the adaptive cut such that $C_{j}\left(0\right)=c$, $C_{j}\left(1\right)\in \partial \Omega$ and $C_{j}\left(u\right)=p_{j}$, for all 1≤j≤J.

Basically, an adaptive cut $C_{j}$ is a continuous curve, and in our work this curve is generated as the concatenation of two minimal paths. In other words, we suppose that

(31) $$C_{j}=C_{1,j}⋓C_{2,j}$$

where $C_{1,j},\;C_{2,j}:\left[0,1\right]\to \Omega$ are two open curves. The operator ⋓ is used for curve concatenation [33,34,35] such that

(32) $$\left(\gamma_{1}⋓\gamma_{2}\right)\left(u\right)=\left\{\begin{array}{cc} \gamma_{1}\left(2u\right), & \mathrm{if}\;0\leq u\leq \frac{1}{2},\\ \gamma_{2}\left(2u-1\right), & \mathrm{if}\;\frac{1}{2}\leq u\leq 1. \end{array}\right.$$

where $\gamma_{1},\;\gamma_{2}:\left[0,1\right]\to \Omega$ are two curves.

Now, we describe how to compute the curves $C_{1,j}$ and $C_{2,j}$. In our work, we compute both paths using the Euler–Mumford elastica model [36,37], such that the path curvature can be taken into account. Basically, the energy of this elastica model can be formulated as:

(33) $$\mathrm{Length}\left(\gamma \right):=\int_{0}^{1}\phi \left(\gamma \left(u\right),\gamma^{\prime }\left(u\right)/∥\gamma^{\prime }\left(u\right)∥\right)\left(1+\tau^{2}\kappa {\left(u\right)}^{2}\right)du$$

where $\phi :\Omega \times S^{1}\to R^{+}$ is a cost function with $S^{1}=\left[0,2\pi \right]$ being an interval of periodic boundary condition, κ:[0,1]→R is the curvature of the regular path γ:[0,1]→Ω, and τ>0 is a parameter that controls the relative importance of the path curvature κ. The cost function ϕ is dependent on the direction $n_{\theta }=\left(-\sin\theta ,\cos\theta \right)$ for any angle $\theta \in S^{1}$. In this work, we compute the cost ϕ using the image gradient-related matrix *W* defined in Equation (25). Let μ>0 be a weight parameter and the cost ϕ be estimated by

(34) $$\phi \left(x,\theta \right)=\exp(-\mu g\left(x,\theta \right)/∥g\left(x,\theta \right){∥}_{\infty })$$

where the function *g* is defined as $g\left(x,\theta \right)=\langle n_{\theta },W\left(x\right)n_{\theta }\rangle$.

This is implemented in two steps. The first step is to compute $C_{1,j}$, which is treated as a curvature-penalized minimal path that globally minimizes the elastica energy under a specified condition

(35) $$\underset{\gamma }{\inf}\;\mathrm{Length}\left(\gamma \right),\;\mathrm{subject}\;\mathrm{to}\;\left\{\begin{array}{c} \gamma (0)=c,\\ \gamma \left(1\right)=p_{j}. \end{array}\right.$$

We adopt the method of orientation lifting, as presented in [36,37], to solve the minimization of the length (33), yielding the minimal path $C_{1,j}$ such that $C_{1,j}\left(0\right)=c$ and $C_{1,j}\left(1\right)=p_{j}$.

Secondly, we apply the similar procedure to generate the the minimal path $C_{1,j}$ obeying that $C_{2,j}\left(0\right)=p_{j}$ and $C_{2,j}\left(1\right)\in \partial \Omega$. This is performing by solving

(36) $$\min_{x\in \partial \Omega }\underset{\gamma }{\inf}\;\mathrm{Length}\left(\gamma \right),\;\mathrm{subject}\;\mathrm{to}\;\left\{\begin{array}{c} \gamma \left(0\right)=p_{j},\\ \gamma (1)=x. \end{array}\right.$$

The target adaptive cut $C_{j}$ can be generated using equation (31). Repeating this procedure, we eventually obtain a series of adaptive cuts $\left\{C_{j}\right\}$, 1≤j≤J.

An example of the construction of multiple adaptive cuts, with the number of vertices set to five, is illustrated in Figure 3b.

### 3.3. Circular Geodesic Voting with Distance Competition and Multiple Cuts

First of all, we construct a set of farthest points

$$Q=\{q_{k}\in \Omega \;|\;1\leq k\leq K\}$$

which are usually taken as the endpoints for computing the voting paths. Note that in the circular geodesic voting method, the user is required to provide the contrast prior to the image gray levels. More precisely, the gray levels at most of the points inside the target region are supposed to be locally higher than the background. In order to remove this constraint and to facilitate the segmentation, we introduce a geodesic distance competition procedure which is able to identify locally the contrast of image gray levels, providing that the target boundaries are unknown.

Recall that each landmark point $p_{j}$ is assigned with an adaptive cut $C_{j}$ whose unit normal vectors are denoted by $N_{j}\left(u\right)\propto M\left(\pi /2\right)C_{j}^{\prime }\left(u\right)$, where M(π/2) is the clockwise rotation matrix with angle π/2 and ∝ is the positively proportional operator. In this case, each landmark point $p_{j}$ corresponds to two offset points $p_{j}^{l}=p_{j}-N_{j}\left(u\right)\epsilon$ and $p_{j}^{r}=p_{j}+N_{j}\left(u\right)\epsilon$, where ϵ>0 is a sufficiently small constant. As a consequence, the point $p_{j}^{l}$ (resp. $p_{j}^{r}$) is placed at the left (resp. right) side of the adaptive cut $C_{j}$.

#### 3.3.1. The Construction of Voting Paths

In the CGV model, two asymmetric quadratic metrics $F_{AQ}^{0}$ (12) and $F_{AQ}^{1}$ (13) are adopted to track minimal paths in counter-clockwise and clockwise directions. This paper considers two sets, $\Pi_{j}^{r}$ and $\Pi_{j}^{l}$, as the search spaces for minimal paths with respect to $p_{j}$:

$$\begin{array}{cc} \; & \Pi_{j}^{r}:=\left\{\gamma \in \mathrm{Lip}\left(\left[0,1\right],\Omega \right)\;|\;\gamma \left(0\right)=p_{j}^{r},\forall u\in \left[0,1\right],\gamma \left(u\right)\notin C_{j}\right\},\\ \; & \Pi_{j}^{l}:=\left\{\gamma \in \mathrm{Lip}\left(\left[0,1\right],\Omega \right)\;|\;\gamma \left(0\right)=p_{j}^{l},\forall u\in \left[0,1\right],\gamma \left(u\right)\notin C_{j}\right\}. \end{array}$$

In other words, the set $\Pi_{j}^{r}$ involves all the paths that emanate from $p_{j}^{r}$. Given an asymmetric quadratic metric, the geodesic distance between two points is the minimal length of an admissible curve γ. By this definition, we construct two geodesic distance maps $D_{j}^{0,r}$ and $D_{j}^{1,r}$:

(37) $$\begin{array}{cc} \; & D_{j}^{0,r}\left(x\right)=\inf\;\left\{\int_{0}^{1}F_{\mathrm{AQ}}^{0}\left(\gamma \left(u\right),\gamma^{\prime }\left(u\right)\right)du\;|\;\gamma \in \Pi_{j}^{r},\;\gamma \left(1\right)=x\right\} \end{array}$$

(38) $$\begin{array}{cc} \; & D_{j}^{1,r}\left(x\right)=\inf\;\left\{\int_{0}^{1}F_{\mathrm{AQ}}^{1}\left(\gamma \left(u\right),\gamma^{\prime }\left(u\right)\right)du\;|\;\gamma \in \Pi_{j}^{r},\;\gamma \left(1\right)=x\right\}. \end{array}$$

In the eikonal PDE framework [32,38,39], the distances maps $D_{j}^{0,r}\left(x\right)$ and $D_{j}^{1,r}\left(x\right)$ are the viscosity solutions to the following PDEs

(39) $$\left\{\begin{array}{cc} \max_{\dot{x}\neq 0}\frac{\langle \nabla D_{j}^{0,r}\left(x\right),\dot{x}\rangle }{F_{\mathrm{AQ}}^{0}\left(x,\dot{x}\right)}=1, & \forall x\neq p_{j}^{r}\\ D_{j}^{0,r}\left(p_{j}^{r}\right)=0. \end{array}\right.\;\mathrm{and}\;\left\{\begin{array}{cc} \max_{\dot{x}\neq 0}\frac{\langle \nabla D_{j}^{1,r}\left(x\right),\dot{x}\rangle }{F_{\mathrm{AQ}}^{1}\left(x,\dot{x}\right)}=1, & \forall x\neq p_{j}^{r}\\ D_{j}^{1,r}\left(p_{j}^{r}\right)=0. \end{array}\right.$$

The geodesic distance map $D_{j}^{0,r}$ (resp. $D_{j}^{0,l}$) corresponds to a set of minimal paths ${\left\{G_{k}^{0,r}\right\}}_{1\leq k\leq K}$ (resp. ${\left\{G_{k}^{1,r}\right\}}_{1\leq k\leq K}$), satisfying that $G_{k}^{0,r}\left(1\right)=q_{k}$ (resp. $G_{k}^{1,r}\left(1\right)=q_{k}$) where $q_{k}\in Q$ is a sampled point. In other words, each sampled point $q_{k}$ leads to two minimal paths, and our goal is to choose a proper one so as to compute the voting scores. For this purpose, we estimate a binary-valued map $b_{r}:\Omega \to \left\{0,1\right\}$ such that

(40) $$b_{r}\left(x\right)=\left\{\begin{array}{cc} 1, & \mathrm{if}\;D_{j}^{1,r}\left(x\right)<D_{j}^{0,r}\left(x\right),\\ 0, & \mathrm{otherwise}. \end{array}\right.$$

Let $G_{k}^{r}$ be the voting path such that $G_{k}^{r}\left(0\right)=p_{j}^{r}$ and $G_{k}^{r}\left(1\right)=q_{k}$. Using the map $b_{r}$, one can identify $G_{k}^{r}$ as follows:

(41) $$G_{k}^{r}=\left\{\begin{array}{cc} G_{k}^{1,r}, & \mathrm{if}\;b_{r}\left(x\right)=1,\\ G_{k}^{0,r}, & \mathrm{otherwise}. \end{array}\right.$$

Similar to the construction of $G_{k}^{r}$, one can obtain the voting paths $G_{k}^{k}$ obeying that $G_{k}^{l}\left(0\right)=p_{j}^{l}$ and $G_{k}^{l}\left(1\right)=q_{k}$. Note that the binary map $b_{l}$ for identifying the voting paths can be simply set to $b_{l}=b_{r}$ or can be computed by Equation (40) using the geodesic distance maps $D_{j}^{0,l}$ and $D_{j}^{1,l}$. Finally, we can also obtain *K* voting paths $G_{k}^{l}$. In Figure 3d, we illustrate the voting paths originating from the farthest points for illustration.

#### 3.3.2. Voting Score Map with Multiple Cuts

For each pair of points $p_{j}^{r}$ and $p_{j}^{l}$, one has two sets of voting paths ${\left\{G_{k}^{r}\right\}}_{1\leq k\leq K}$ and ${\left\{G_{k}^{l}\right\}}_{1\leq k\leq K}$. Using the geodesic voting method introduced in the section on “Section Performing Geodesic Voting”, we can compute a voting score map $V_{j}:\Omega \to R^{+}$ using (23) by setting the parameter α=0. This means that we only use the circular path voting part. As illustrated in the example in Figure 3e, the final voting score map V can be simply set as the summation of all the $V_{j}$:

(42) $$V\left(x\right)=\sum_{j=1}^{J}V_{j}\left(x\right).$$

## 4. Experiments

In this section, we conduct experiments to compare the proposed multi-source circular geodesic voting model with both deep learning and geodesic voting methods through qualitative and quantitative analyses.

### 4.1. Experimental Configuration

#### 4.1.1. Datasets

We employ both natural and medical images in our experiments to assess the performance of the methods.

- *Natural Images:* We utilize the Berkeley Segmentation Dataset 500 (BSDS500) [40] as the dataset for natural images, which serves as a standard benchmark for contour detection. This dataset is specifically designed to evaluate natural edge detection, encompassing not only object contours but also interior and background boundaries.
- *Medical Images:* For medical images, we select X-ray computed tomography (CT) scans from [41]. This dataset is commonly used in medical image segmentation evaluations, with image resolution ranging from 100×100 to 150×150 pixels, depending on the specific context of each test.

For both datasets, we select 80% of the images as the training set for deep learning-based methods, with the remaining 20% used as the test set for performance evaluation.

#### 4.1.2. Baseline Methods

The baseline methods include the deep learning-based PolarMask [13] and the geodesic voting-based CGV [24]. For PolarMask, the training process follows that in [13], where ResNet101 and ResNeXt101 serve as the backbone networks, and the number of rays is set to 36. For CGV, the parameter settings align with those in [24]. Specifically, the number of endpoints *N* is set to 1000, and the parameters α and β used to compute the final voting score in Equation (23) are both set to 1. The threshold value for the final segmentation result is set to 100.

### 4.2. Qualitative Comparison Analysis

**Figure 4:**
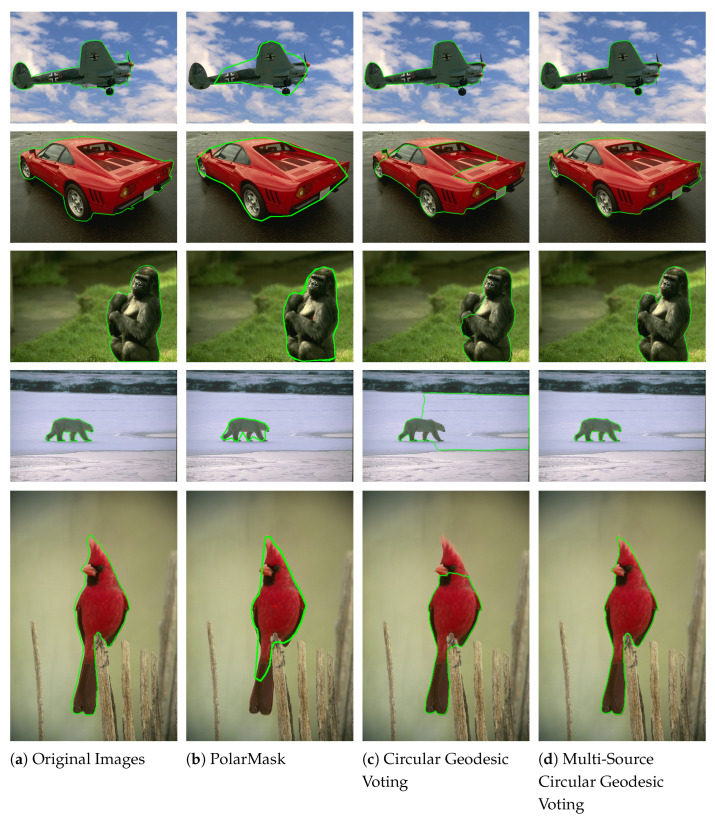
Qualitative comparison results on nature images, where the green lines denote the segmentation contours. Column 1 displays the original images with ground truth segmentation contours indicated by green lines. Columns 2–4 show the segmented results produced by the PolarMask, circular geodesic voting, and multi-source circular geodesic voting methods, respectively.

Figure 4 presents qualitative comparison results on natural images. The green lines denote the segmentation contours. In the original images (Column 1), target objects are surrounded by complex backgrounds, have intricate shapes, and exhibit intensity inhomogeneity. Additionally, some target boundaries are not clearly defined, making the segmentation task challenging. The PolarMask method (Column 2) struggles to capture the detailed contours of objects when boundaries are complex, as seen in the images in the second and fourth rows. It may also miss essential object components due to the limited number of points on the contour, as observed in the images in the first and fifth rows. The circular geodesic voting method (Column 3) encounters the “short-cut” problem when the object contains intricate textures, as evident in the images in the first, second, third, and fifth rows. This method is also influenced by strong background shapes because it uses only a single source point to compute the geodesic distance map. In contrast, the proposed multi-source circular geodesic voting method (Column 4) overcomes these limitations, accurately detecting target boundaries even in complex scenarios.

**Figure 5:**
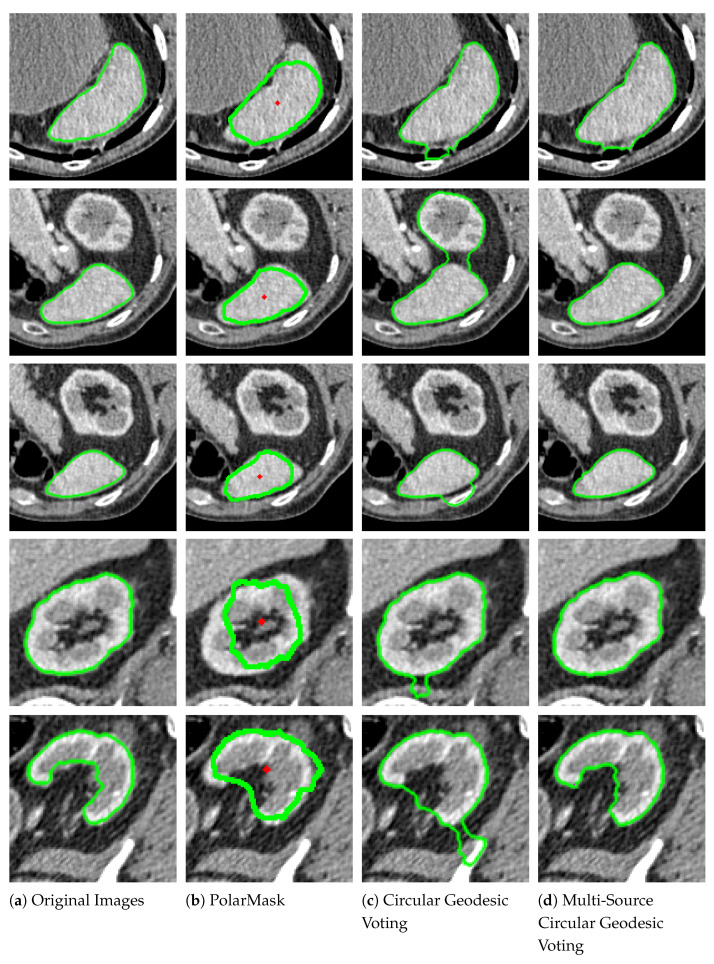
Qualitative comparison results on CT images. The green lines denote the segmentation contours. Column 1 displays the original images. Columns 2–4 show the segmented results produced by the PolarMask, circular geodesic voting, and multi-source circular geodesic voting methods, respectively.

Figure 5 illustrates qualitative comparison results on medical CT images. In the first column, the original images highlight the challenges of segmenting the liver contour due to its low contrast with adjacent tissues, which complicates boundary differentiation. The liver’s irregular shape and texture, along with the partial volume effect, result in blurred edges that further complicate segmentation. Given the difficulty of this task, the PolarMask method (Column 2) fails to capture a complete set of contour points around the liver. The circular geodesic voting method (Column 3) is affected by inherent noise along the liver boundary and artifacts from nearby structures, such as the ribs or diaphragm, leading to incorrect areas being included in the contour. By contrast, the proposed multi-source circular geodesic voting method (Column 4) demonstrates robustness against noise and accurately delineates the target boundaries.

### 4.3. Quantitative Performance Analysis

To compare the methods rigorously and convincingly, we conduct quantitative comparisons using the Dice score J, chosen for its effectiveness in measuring the overlap between predicted paths and ground truth regions [15,24]. The accuracy of the tested methods is measured by the Dice score, defined as follows:

(43) $$J=\frac{\#|S\cap G|}{\#|S|},$$

where *S* represents the set of grid points traversed by the evaluated paths, *G* denotes the region corresponding to the ground truth, and #|S| indicates the number of elements in the set *S*. The accuracy score J ranges within the interval [0,1], with higher values of J indicating better performance.

To quantitatively evaluate the segmentation accuracy of the proposed model, we perform a comparative analysis of different methods on the test set of the CT image dataset. Table 1 presents the quantitative comparison results for the PolarMask, CGV, and PolarVoting methods on the test set. These metrics report the mean accuracy and standard deviation of segmentation performance across the dataset.

From the results in Table 1, it is evident that the PolarVoting method achieves a significantly higher mean accuracy (0.8561) compared to both PolarMask (0.2928) and single-source CGV (0.6639), with a notably lower standard deviation (0.1541) than that of the single-source CGV method (0.3344). This suggests that the PolarVoting approach provides more consistent segmentation outcomes.

**Figure 6:**
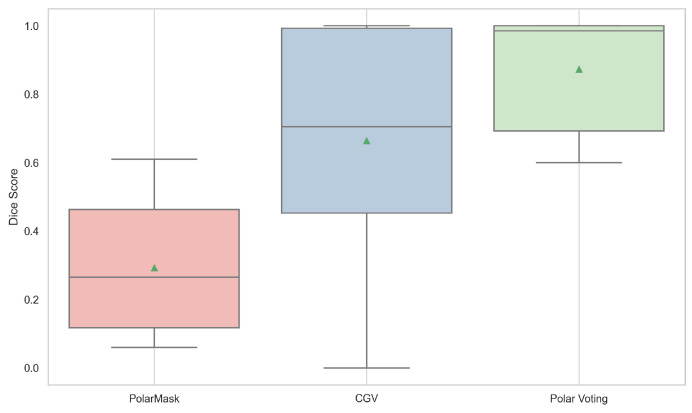
Box plots of the Dice scores for the PolarMask, circular geodesic voting, and multi-source circular geodesic voting methods on the test set of CT images. The green triangles represent the mean Dice score.

In contrast, PolarMask demonstrates the lowest performance in both mean accuracy and standard deviation, indicating insufficient robustness and precision for CT image segmentation in this context. While the single-source CGV method outperforms PolarMask, it remains inferior to the proposed PolarVoting approach in terms of both accuracy and consistency. The box plots of the Dice scores in Figure 6 further support these findings.

### 4.4. Influence of Source Points on Performance

#### 4.4.1. Effect of the Number of Source Points

**Figure 7:**
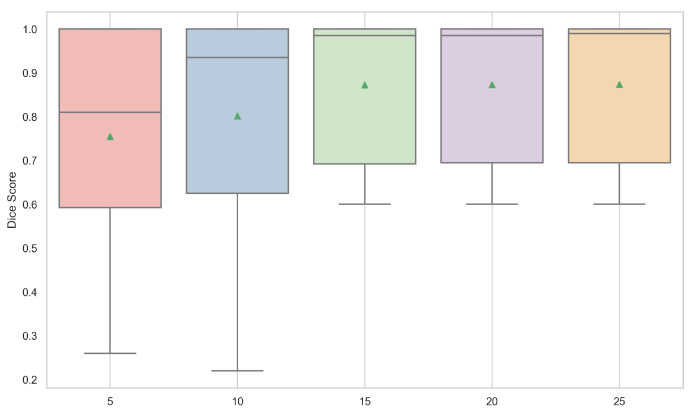
Box plots of the Dice scores for different numbers of source points for multi-source circular geodesic voting methods on the test set of CT images. The green triangles represent the mean Dice score.

Table 2 and Figure 7 present the mean and standard deviation of the Dice scores, along with bar plots showing the Dice scores for different numbers of source points in the multi-source circular geodesic voting method on the test set of CT images.

From these results, we observe that the Dice score improves as the number of source points increases from 5 to 25. Specifically, the mean Dice score rises from 0.7550 with 5 source points to 0.8739 with 25 points. This trend suggests that a higher number of source points enhances segmentation performance in the multi-source circular geodesic voting approach, likely due to the additional spatial information provided by multiple source points, allowing for more accurate alignment of geodesic votes within the CT image domain.

Furthermore, the standard deviation of the Dice scores decreases slightly as the number of source points exceeds 10. For example, with 5 and 10 source points, the Std is relatively high, around 0.243, indicating greater variability in performance across different samples in the test set. However, from 15 source points onward, the Std reduces to approximately 0.157, indicating that the method’s performance becomes more consistent as the number of source points increases. This increased stability can be attributed to a more robust spatial representation achieved with a higher number of source points, which helps to minimize performance fluctuations.

The box plots in Figure 7 further illustrate the distribution of Dice scores across different configurations. With five source points, the Dice score distribution shows greater variability, including a wider range with lower minimum values. In contrast, the distributions become more compact and centered around higher median values as the number of source points increases, particularly from 15 to 25. This indicates that the voting-based method performs more reliably and effectively with an increased number of source points, leading to improved segmentation accuracy.

**Figure 8:**
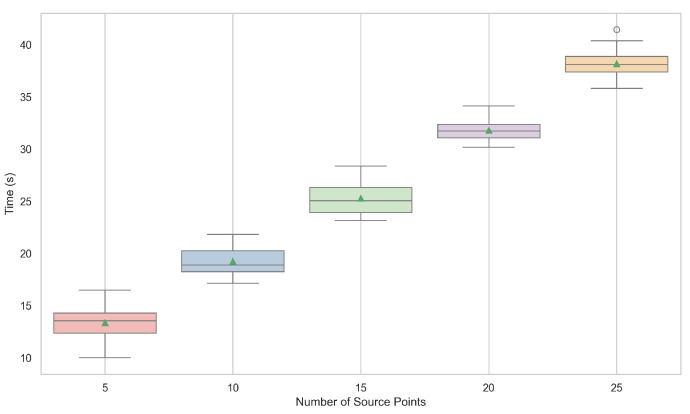
Box plots of the execution time for different numbers of source points for multi-source circular geodesic voting methods on the test set of CT Images. The green triangles represent the mean execution time.

Figure 8 illustrates the execution time of the PolarVoting model as a function of the number of source points. The results indicate that the execution time increases linearly with the number of source points. This linear relationship suggests that the computational complexity of the PolarVoting model is directly influenced by the number of source points used in the segmentation process. While a higher number of source points may improve segmentation accuracy by better approximating the target boundary, it also increases the computational burden. This trade-off between execution time and accuracy highlights the importance of selecting an appropriate number of source points based on application-specific requirements.

#### 4.4.2. Effect of the Placement of Source Points

In the PolarVoting model, the initial placement of source points is determined using PolarMask, and their locations are subsequently updated to align with the nearby target boundary. To evaluate the impact of the initial placement of source points, a PolarMask model is trained to generate 36 source points. A fixed number of source points, specifically 5, 10, and 15, are then sampled using different random seeds. This procedure is repeated 10 times for each configuration.

**Figure 9:**
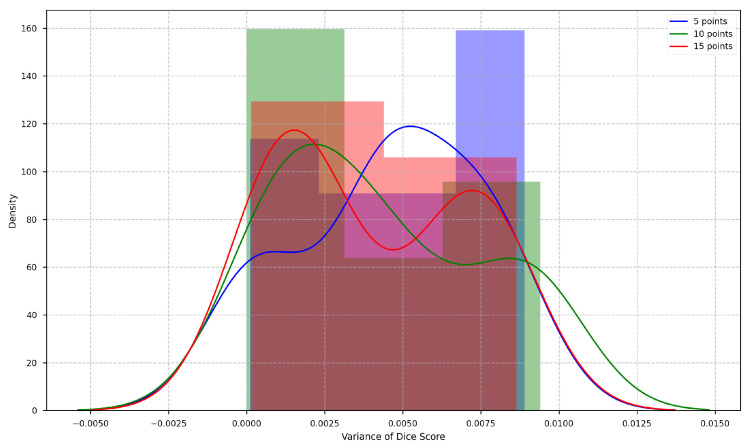
Variance distribution of Dice scores for different initial placements of source points in the PolarVoting model on the test set of CT images.

For each image in the test set of the CT image dataset, the Dice score is computed, and the variance across the 10 iterations is calculated. The distribution of these variances is depicted in Figure 9. The results indicate that the variance distributions for different numbers of source points are similar and remain small. This demonstrates that the location update strategy employed by the PolarVoting model ensures stable performance, rendering it insensitive to the initial placement of source points.

### 4.5. Performance Under Adverse Conditions

In this section, the performance of the proposed multi-source CGV model is evaluated under adverse conditions, including images affected by noise, blurring, and poor brightness.

**Figure 10:**
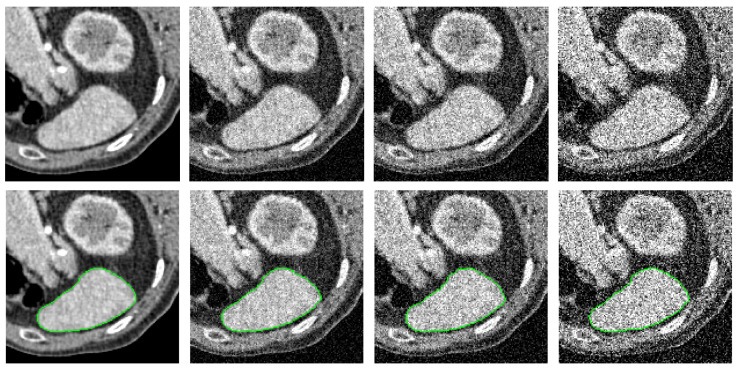
Performance of the PolarVoting model under noise conditions on CT images. The green lines represent the segmentation contours. Column 1 shows the original image alongside its segmentation result. Columns 2–4 present images affected by Gaussian noise with variances of 0.01, 0.02, and 0.05, respectively, along with the corresponding segmentation results.

**Figure 11:**
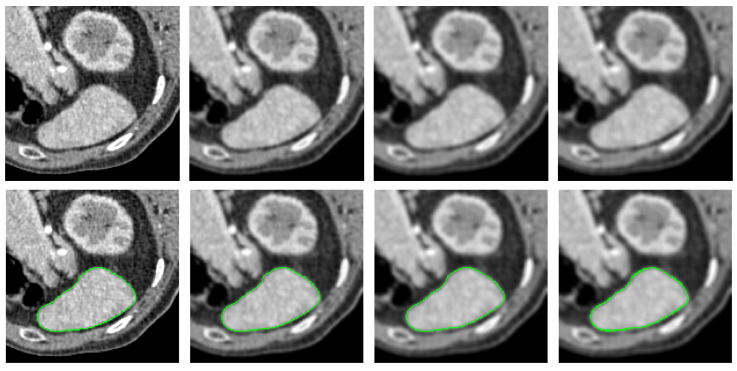
Performance of the PolarVoting model under blur conditions on CT images. The green lines represent the segmentation contours. Column 1 shows the original image alongside its segmentation result. Columns 2–4 display images with Gaussian blur levels of σ=1, σ=2, and σ=3, respectively, along with the corresponding segmentation results.

**Figure 12:**
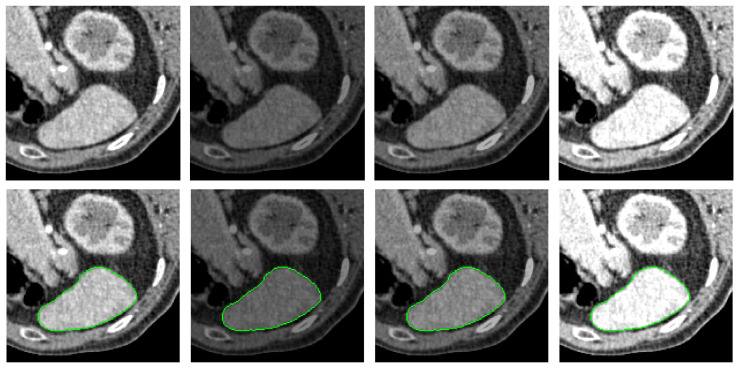
Performance of the PolarVoting model under brightness variations on CT images. The green lines represent the segmentation contours. Column 1 shows the original image alongside its segmentation result. Columns 2–4 display images with brightness adjustment factors of 0.5, 0.7, and 1.3, respectively, along with the corresponding segmentation results.

Figure 10, Figure 11 and Figure 12 present the segmentation results of PolarVoting under various adverse conditions. For noise conditions, Gaussian noise with variances of 0.01, 0.02, and 0.05 is added to the images. For blur conditions, the images are blurred using a rotationally symmetric Gaussian low-pass filter with σ values of 1, 2, and 3. For brightness variations, the brightness of the images is adjusted by factors of 0.2, 0.5, and 1.3, where values less than 1 darken the image and values greater than 1 brighten it. The segmentation results demonstrate that the PolarVoting model is robust to noise, blur, and brightness variations. It effectively tracks the correct boundaries even in the presence of significant noise, severe blurring, or extremely low brightness.

## 5. Conlusions

In this paper, we introduce a novel multi-source circular geodesic voting model for image segmentation, which effectively integrates the geometric regularization of traditional voting models which can take advantage of the adaptive representation capabilities of deep learning, particularly when carried out by the classical PolarMask model. By employing multiple source points for constructing the voting score map, the proposed model overcomes the limitations of single-source CGV approaches, especially when dealing with complex and noisy images in many segmentation tasks. Experimental results on both natural and medical image datasets demonstrate that the multi-source CGV model achieves superior accuracy and robustness compared to existing methods, such as PolarMask and single-source CGV, validating its potential for diverse image segmentation applications. Future work will be devoted to automated selection of source points and further optimization of the model for large-scale segmentation tasks, aiming to enhance both efficiency and accuracy across broader application domains.

## Figures and Tables

**Table 1:** The quantitative comparison results of the PolarMask, circular geodesic voting, and multi-source circular geodesic voting methods on the test set of CT images.

PolarMask		CGV		PolarVoting	
**Mean**	**Std**	**Mean**	**Std**	**Mean**	**Std**
0.2928	0.1841	0.6639	0.3344	0.8561	0.1541

**Table 2:** The quantitative comparison results of different numbers of source points for multi-source circular geodesic voting methods on the test set of CT images.

Number of Source Points									
**5**		**10**		**15**		**20**		**25**	
**Mean**	**Std**	**Mean**	**Std**	**Mean**	**Std**	**Mean**	**Std**	**Mean**	**Std**
0.7550	0.2432	0.8017	0.2436	0.8728	0.1576	0.8733	0.1572	0.8739	0.1576

## Data Availability

The data used to support the findings of this study are available from the corresponding author upon request.

## References

[1] Luo X., Wang G., Liao W., Chen J., Song T., Chen Y., Zhang S., Metaxas D.N., Zhang S. (2022). Semi-supervised medical image segmentation via uncertainty rectified pyramid consistency. Med. Image Anal..

[2] Ning Z., Zhong S., Feng Q., Chen W., Zhang Y. (2021). SMU-Net: Saliency-guided morphology-aware U-Net for breast lesion segmentation in ultrasound image. IEEE Trans. Med. Imaging.

[3] Zhou W., Dong S., Lei J., Yu L. (2022). MTANet: Multitask-aware network with hierarchical multimodal fusion for RGB-T urban scene understanding. IEEE Trans. Intell. Veh..

[4] Yao S., Guan R., Huang X., Li Z., Sha X., Yue Y., Lim E.G., Seo H., Man K.L., Zhu X. (2023). Radar-camera fusion for object detection and semantic segmentation in autonomous driving: A comprehensive review. IEEE Trans. Intell. Veh..

[5] Deng Y., Xi H., Zhou G., Chen A., Wang Y., Li L., Hu Y. (2023). An effective image-based tomato leaf disease segmentation method using MC-UNet. Plant Phenomics.

[6] Wu F., Yang Z., Mo X., Wu Z., Tang W., Duan J., Zou X. (2023). Detection and counting of banana bunches by integrating deep learning and classic image-processing algorithms. Comput. Electron. Agric..

[7] Minaee S., Boykov Y., Porikli F., Plaza A., Kehtarnavaz N., Terzopoulos D. (2021). Image segmentation using deep learning: A survey. IEEE Trans. Pattern Anal. Mach. Intell..

[8] Wang Z., Wang E., Zhu Y. (2020). Image segmentation evaluation: A survey of methods. Artif. Intell. Rev..

[9] Sultana F., Sufian A., Dutta P. (2020). Evolution of image segmentation using deep convolutional neural network: A survey. Knowl.-Based Syst..

[10] Ronneberger O., Fischer P., Brox T. (2015). U-net: Convolutional networks for biomedical image segmentation. Proceedings of the Medical Image Computing and Computer-Assisted Intervention—MICCAI 2015: 18th International Conference.

[11] Chen L.C., Papandreou G., Kokkinos I., Murphy K., Yuille A.L. (2017). Deeplab: Semantic image segmentation with deep convolutional nets, atrous convolution, and fully connected crfs. IEEE Trans. Pattern Anal. Mach. Intell..

[12] Xie E., Sun P., Song X., Wang W., Liu X., Liang D., Shen C., Luo P. Polarmask: Single shot instance segmentation with polar representation. Proceedings of the IEEE/CVF Conference on Computer Vision and Pattern Recognition.

[13] Xie E., Wang W., Ding M., Zhang R., Luo P. (2021). Polarmask++: Enhanced polar representation for single-shot instance segmentation and beyond. IEEE Trans. Pattern Anal. Mach. Intell..

[14] Zou L., Song L.T., Weise T., Wang X.F., Huang Q.J., Deng R., Wu Z.Z. (2021). A survey on regional level set image segmentation models based on the energy functional similarity measure. Neurocomputing.

[15] Liu L., Chen D., Shu M., Cohen L.D. (2024). Grouping Boundary Proposals for Fast Interactive Image Segmentation. IEEE Trans. Image Process..

[16] Yang D., Peng B., Al-Huda Z., Malik A., Zhai D. (2022). An overview of edge and object contour detection. Neurocomputing.

[17] Heidler K., Mou L., Loebel E., Scheinert M., Lefèvre S., Zhu X.X. (2023). A deep active contour model for delineating glacier calving fronts. IEEE Trans. Geosci. Remote Sens..

[18] Choi J., Suh D. (2025). A depthwise convolutional neural network model based on active contour for multi-defect wafer map pattern classification. Eng. Appl. Artif. Intell..

[19] Xu C., Wang J., Tao J., Zhang J., Zheng P. (2022). A knowledge augmented deep learning method for vision-based yarn contour detection. J. Manuf. Syst..

[20] Li Y., Cao G., Wang T., Cui Q., Wang B. (2020). A novel local region-based active contour model for image segmentation using Bayes theorem. Inf. Sci..

[21] Nouri M., Baleghi Y. (2023). An active contour model reinforced by convolutional neural network and texture description. Neurocomputing.

[22] Nguyen D.H., Nguyen D.M., Mai T.T., Nguyen T., Tran K.T., Nguyen A.T., Pham B.T., Nguyen B.T. (2022). ASMCNN: An efficient brain extraction using active shape model and convolutional neural networks. Inf. Sci..

[23] Biesok M., Juszczyk J., Badura P. (2024). Breast tumor segmentation in ultrasound using distance-adapted fuzzy connectedness, convolutional neural network, and active contour. Sci. Rep..

[24] Zhou S., Liu H., Liu L., Shu M., Chen D., Cohen L.D. (2024). A Generalized Geodesic Voting Framework for Interactive Image Segmentation. IEEE Trans. Instrum. Meas..

[25] Tian Z., Chu X., Wang X., Wei X., Shen C. (2022). Fully convolutional one-stage 3d object detection on lidar range images. Adv. Neural Inf. Process. Syst..

[26] Lions P.L. (1982). Generalized Solutions of Hamilton-Jacobi Equations.

[27] Sethian J.A., Vladimirsky A. (2003). Ordered upwind methods for static Hamilton–Jacobi equations: Theory and algorithms. SIAM J. Numer. Anal..

[28] Mirebeau J.M. (2014). Anisotropic fast-marching on cartesian grids using lattice basis reduction. SIAM J. Numer. Anal..

[29] Peyré G., Cohen L.D. (2006). Geodesic remeshing using front propagation. Int. J. Comput. Vis..

[30] Sochen N., Kimmel R., Malladi R. (1998). A general framework for low level vision. IEEE Trans. Image Process..

[31] Xu C., Prince J.L. (1998). Snakes, shapes, and gradient vector flow. IEEE Trans. Image Process..

[32] Mirebeau J.M., Portegies J. (2019). Hamiltonian fast marching: A numerical solver for anisotropic and non-holonomic eikonal PDEs. Image Process. Line.

[33] Mille J., Bougleux S., Cohen L.D. (2015). Combination of piecewise-geodesic paths for interactive segmentation. Int. J. Comput. Vis..

[34] Chen D., Zhu J., Zhang X., Shu M., Cohen L.D. (2021). Geodesic paths for image segmentation with implicit region-based homogeneity enhancement. IEEE Trans. Image Process..

[35] Chen D., Mirebeau J.M., Shu H., Cohen L.D. (2024). A Region-based Randers geodesic approach for image segmentation. Int. J. Comput. Vis..

[36] Chen D., Mirebeau J.M., Cohen L.D. (2017). Global minimum for a Finsler elastica minimal path approach. Int. J. Comput. Vis..

[37] Mirebeau J.M. (2018). Fast-marching methods for curvature penalized shortest paths. J. Math. Imag. Vis..

[38] Chen D., Spencer J., Mirebeau J.M., Chen K., Cohen L.D. Asymmetric geodesic distance propagation for active contours. Proceedings of the 29th British Machine Vision Conference (BMVC’18).

[39] Cohen L.D., Kimmel R. (1997). Global minimum for active contour models: A minimal path approach. Int. J. Comput. Vis..

[40] Martin D., Fowlkes C., Tal D., Malik J. A Database of Human Segmented Natural Images and its Application to Evaluating Segmentation Algorithms and Measuring Ecological Statistics. Proceedings of the Eighth IEEE International Conference on Computer Vision.

[41] Spencer J., Chen K., Duan J. (2018). Parameter-free selective segmentation with convex variational methods. IEEE Trans. Image Process..

